# Epitope(s) involving amino acids of the fusion loop of Japanese encephalitis virus envelope protein is(are) important to elicit protective immunity

**DOI:** 10.1128/jvi.01773-23

**Published:** 2024-03-26

**Authors:** Yi-Chin Fan, Jo-Mei Chen, Yi-Ying Chen, Yuan-Dun Ke, Gwong-Jen J. Chang, Shyan-Song Chiou

**Affiliations:** 1 Institute of Epidemiology and Preventive Medicine, College of Public Health, National Taiwan University, Taipei, Taiwan; 2 Master of Public Health Degree Program, College of Public Health, National Taiwan University, Taipei, Taiwan; 3 Graduate Institute of Microbiology and Public Health, College of Veterinary Medicine, National Chung Hsing University, Taichung, Taiwan; 4 Arboviral Diseases Branch, Centers for Disease Control and Prevention, Fort, Fort Collins, Colorado, USA; University of North Carolina at Chapel Hill, Chapel Hill, North Carolina, USA

**Keywords:** Japanese encephalitis virus, fusion loop

## Abstract

Dengue vaccine candidates have been shown to improve vaccine safety and efficacy by altering the residues or accessibility of the fusion loop on the virus envelope protein domain II (DII_FL_) in an *ex vivo* animal study. The current study aimed to comprehensively investigate the impact of DII_FL_ mutations on the antigenicity, immunogenicity, and protective efficacy of Japanese encephalitis virus (JEV) virus-like particles (VLPs) in mice. We found the DII_FL_ G106K/L107D (KD) and W101G/G106K/L107D (GKD) mutations altered the binding activity of JEV VLP to cross-reactive monoclonal antibodies but had no effect on their ability to elicit total IgG antibodies in mice. However, JEV VLPs with KD or GKD mutations induced significantly less neutralizing antibodies against JEV. Only 46% and 31% of the KD and GKD VLPs-immunized mice survived compared to 100% of the wild-type (WT) VLP-immunized mice after a lethal JEV challenge. In passive protection experiments, naïve mice that received sera from WT VLP-immunized mice exhibited a significantly higher survival rate of 46.7% compared to those receiving sera from KD VLP- and GKD VLP-immunized mice (6.7% and 0%, respectively). This study demonstrated that JEV DII_FL_ is crucial for eliciting potently neutralizing antibodies and protective immunity against JEV.

**IMPORTANCE:**

Introduction of mutations into the fusion loop is one potential strategy for generating safe dengue and Zika vaccines by reducing the risk of severe dengue following subsequent infections, and for constructing live-attenuated vaccine candidates against newly emerging Japanese encephalitis virus (JEV) or Japanese encephalitis (JE) serocomplex virus. The monoclonal antibody studies indicated the fusion loop of JE serocomplex viruses primarily comprised non-neutralizing epitopes. However, the present study demonstrates that the JEV fusion loop plays a critical role in eliciting protective immunity in mice. Modifications to the fusion loop of JE serocomplex viruses might negatively affect vaccine efficacy compared to dengue and zika serocomplex viruses. Further studies are required to assess the impact of mutant fusion loop encoded by commonly used JEV vaccine strains on vaccine efficacy or safety after subsequent dengue virus infection.

## INTRODUCTION

Flavivirus comprises several serocomplexes, including human-pathogenic viruses such as yellow fever virus (YFV), Japanese encephalitis virus (JEV), West Nile virus (WNV), four serotypes of dengue virus (DENV-1 to −4), zika virus (ZIKV), Powassan virus (POWV), and tick-borne encephalitis virus (TBEV) (1, 2). While vaccines against YFV, JEV, TBEV, and DENV are available for humans (2), recent studies have suggested that genotype III (GIII) JEV and DENV vaccines may reduce vaccine potency against emerging GI JEV (3–7) or increase the risk of hospitalization after the subsequent DENV infection (8), respectively. The mechanisms underlying immune protection and immune pathogenesis in flavivirus infections encompass the responses of both B cells and T cells (9–15). Understanding virus antigens or regions that elicit protective or pathogenic immunity may lead to improve the current vaccines and to rationally develop a safer vaccine candidate.

Flavivirus possesses a single-stranded, positive-sense RNA, which undergoes translation to yield three structural proteins [Capsid (C), precursor membrane protein (prM), and envelope (E) proteins] and seven nonstructural proteins (nonstructural protein 1, 2A, 2B, 3, 4A, 4B, and 5, abbreviated as NS1, NS2A, NS2B, NS3, NS4A, NS4B, and NS5, respectively). Notably, CD4^+^ and CD8^+^ T-cell epitopes have predominantly been identified on structural and nonstructural proteins, respectively (9, 10, 12). The majority of antibody responses recognize the virus E and NS1 proteins (9, 16). E protein is the primary target to elicit neutralizing antibodies correlated with vaccine potency (17–19). The E protein comprises three domains (DI, DII, and DIII) and plays a critical role in the binding of virus to cellular receptors and viral entry. Most potently neutralizing monoclonal antibodies (mAbs) recognized DIII and E dimer epitopes (EDE), whereas poor or non-neutralizing mAbs mainly targeted fusion loop (residues 98–110) on DII (DII_FL_), which were involved in enhancing dengue disease severity (11, 16). However, flavivirus infection predominantly elicited anti-DII_FL_ antibodies rather than anti-DIII antibodies (16, 20). Vaccine candidates using specific antigens or structurally modified antigens can be used to modulate host immunity against virus infection such as human immunodeficiency virus, influenza viruses, and severe acute respiratory syndrome coronavirus 2 (21–23). It has been suggested that flavivirus vaccine candidates should aim to refocus antibody response from targeting DII_FL_ to DIII and EDE epitopes to enhance the neutralizing antibody response and reduce the antibody-dependent enhancement (ADE)-related antibody response, which is involved in developing severe dengue (11, 16).

A DIII-based flavivirus vaccine candidate showed lower immunogenicity and induced less neutralizing antibodies than the soluble E monomer (24). Thus, the vaccine candidate should preserve the neutralizing epitopes on the other domains and/or inter-domains (24). E-homodimer antigens re-directed the antibody response from DII_FL_ to non-DII_FL_ and EDE epitopes and elicited higher protective potency as compared to E monomers (25, 26). This modulation was related to different antigenicity in the quaternary epitopes rather than DII_FL_ epitopes, the result suggested that reducing DII_FL_ immunogenicity might indirectly improve the production of neutralizing antibodies (25, 26). Flavivirus virus-like particles (VLPs) formed a similar structure to virions and were thought to induce more quaternary-dependent antibodies (27, 28). Previous studies introduced mutations into DII_FL_ to reduce its immunodominance and used VLPs or VLP-expressing DNA plasmids as immunogens (29–32). Mutations in DENV and ZIKV DII_FL_ were found to inhibit the occurrence of ADE in immunized mice. However, the impact of these mutations on the DII_FL_ immunogenicity and its ability to elicit neutralizing antibodies remains unclear and inconsistent (31, 32). This inconsistency may be due to different DII_FL_ mutations and variations in the immune landscape of various serocomplex groups. It has been suggested that DII_FL_ of JE serocomplex viruses is not necessary for inducing neutralizing antibodies, as the most potent neutralizing antibodies recognize DIII rather than DII_FL_ (33–36). Some studies have indicated that encoding DII_FL_ mutations could influence the antigenicity of JE serocomplex virus-derived VLPs or the immunogenicity of JEV VLP-expressing plasmids (37–39). However, there is a need to conduct comprehensive research on the impact of DII_FL_ mutations on the antigenicity, immunogenicity, and protective potency of JE serocomplex virus-derived immunogens.

This study aims to investigate the role of DII_FL_ in eliciting B-cell-direct protective immunity against JE serocomplex viruses. The VLP-expressing DNA plasmid has been shown to elicit cytotoxic T-cell immunity (40); thus, we decided to utilize JEV VLP to examine the influence of DII_FL_ mutations on the antigenicity, immunogenicity, and its ability to elicit neutralizing antibodies and evaluated the DII_FL_-mutated immunogen as the next-generation vaccine candidate in mice.

## RESULTS

### Identification of the DII_FL_ epitopes on genotype I (GI) VLPs using flaviviral mAbs

**Fig 1:**
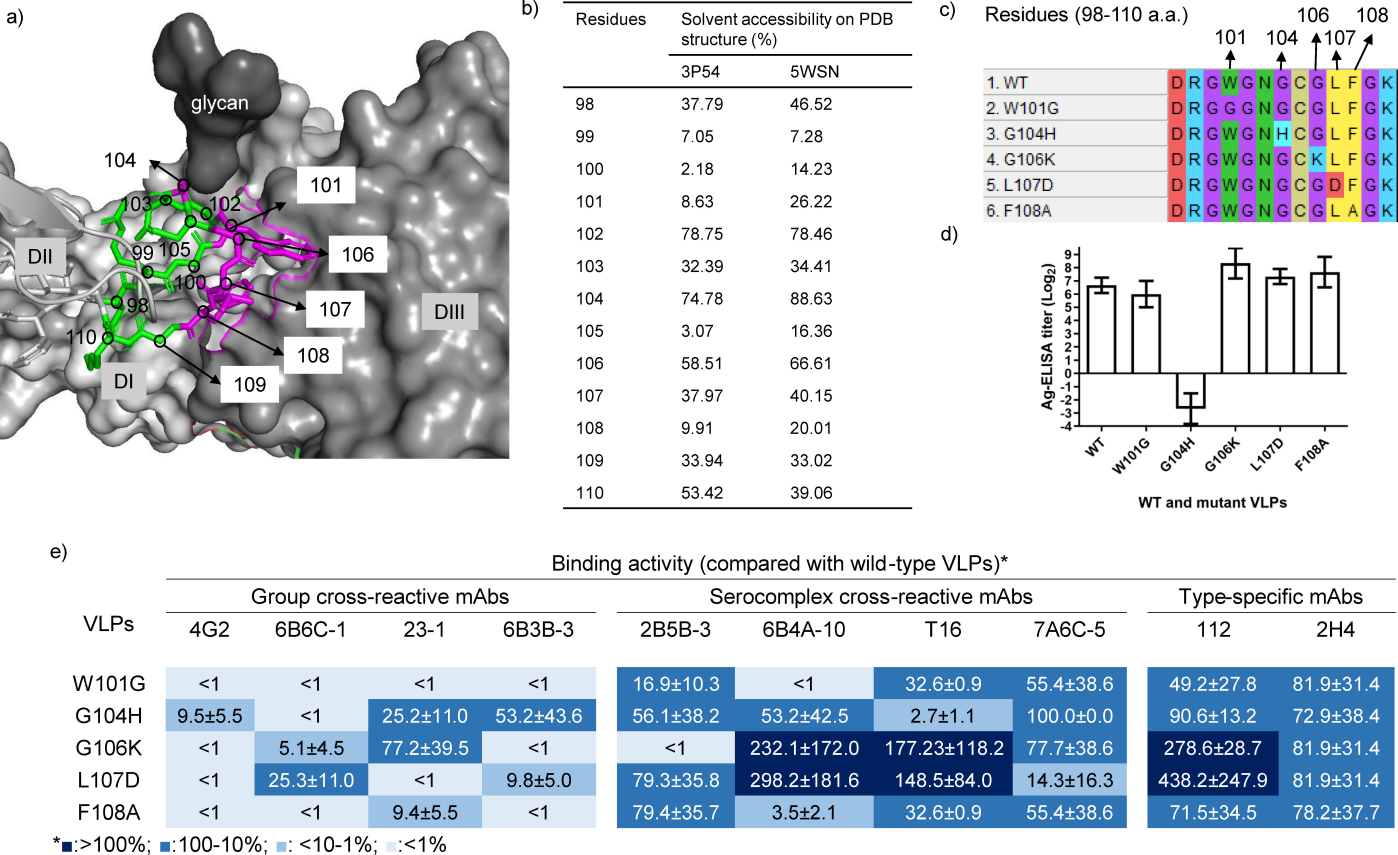
Structural location and contribution of single amino acid residues in the fusion loop to the binding of flaviviral monoclonal antibodies (mAbs). (**a**) SWISS-MODEL (https://swissmodel.expasy.org/) was used to model the E dimers of the JEV GI YL2009-4 virus with a PDB template of 3P54. The structural location of the fusion loop (on the simulated E dimers with 3P54) and the N-linked glycan (adapted from the PDB template of 5N0A) were presented in PyMOL (https://pymol.org/2/). The fusion loop and the mutated residues were colored in green and magenta, respectively. (**b**) Dezyme software (http://www.dezyme.com/) estimated the solvent accessibility of residues on the simulated E-dimer with the 3P54 template and the E-dimer on JEV virion (PDB: 5WSN). (**c**) The fusion loop sequences of pVJGI WT and mutant VLP plasmids encoding W101G, G104H, G106K, L107D, or F108A mutations were presented. (**D**) Genotype I (GI) WT and mutant VLPs were collected from COS-1 cells transfected with the VLP-expressing plasmids. The endpoint titer of the secreted VLPs was measured by antigen-capture enzyme-linked immunosorbent assay (Ag-ELISA). The mean and standard deviation were presented. (**e**) Ag-ELISA was used to measure the interaction of GI WT and mutant VLPs with group cross-reactive, JE serocomplex cross-reactive, and type-specific mAbs. The mAb binding activity against mutant VLPs was compared to WT VLPs and calculated by [Log ^endpoint titer against (mutant VLP／ WT VLP)^] ×100%. The binding activity was presented as mean ± SD and color from deep blue to light blue based on the mean values of ˃100%, 10%–100%, ˂10%–1%, or ˂1%. All experiments were performed in triplicate.

JEV GI VLPs induced cross-neutralizing antibodies against four JEV genotypes and provided cross-protection against GI and GIII viruses in pigs (41). In this study, we used flaviviral mAbs to identify the DII_FL_ epitopes on GI VLPs. Because the structure of VLP was unavailable, we analyzed the conformational location and antibody accessibility of DII_FL_ on a simulated E-dimer structure with GI virus sequence or on an E-dimer structure of GIII JEV virion (PDB: 5WSN) (Fig. 1a and b). The 98 to 110 residues of DII_FL_ were conformationally interfered by DIII and the N-linked glycan at residue 154 on another monomer (Fig. 1a). The 98, 102, 104, 106, 107, and 110 residues had higher accessibility of 35% for antibodies than the other residues on the simulated E-dimer of the GI virus and GIII JEV E dimer (Fig. 1b). The estimated accessibility of residues 100, 101, 105, and 108 varied between the two E-dimer structures. This discrepancy may be linked to the utilization of either recombinant E proteins (PDB: 3P54) or virus particles for structure resolution (PDB: 5WSN). Flavivirus DII_FL_ epitopes were located at residues 101, 104, 106, 107, and 108 but the precise residues remained unknown for GI VLPs (37, 42–44). Flavivirus VLPs encoding W101G, G104H, G106K, L107D, or F108A were secretable and interactive with flavivirus mAbs (43, 44). Thus, we generated mutated GI VLPs with a single DII_FL_ W101G, G104H, G106K, L107D, or F108A mutation (Fig. 1c). Antigen-capture (Ag)-ELISA confirmed the yield of the secreted wild-type (WT) and mutant VLPs were similar except for the G104H VLP, which could only be detected in concentrated culture fluid (Fig. 1d). As compared to WT VLPs, both W101G and F108A VLPs had less than 10% of binding activity against all group cross-reactive mAbs as well as 6B4A-10 mAb. By contrast, G106K and L107D VLPs increased the binding activity to the neutralizing T16 mAb and JEV type-specific 112 mAb (Fig. 1e). We selected G106K and L107D mutations to evaluate the role of DII_FL_ in inducing neutralizing antibodies. We further included the W101G mutation to investigate the contribution of group cross-reactive antibodies to the neutralizing activity since the W101G mutation exhibited more reduction in binding activity with 23–1 and 6B4A-10 mAbs than the F108A mutation. We excluded G104H VLPs in future studies due to the low level of VLP secretion.

### The impact of DII_FL_ G106K/L107D and W101G/G106K/L107D mutations on the particle formation and antigenic activity of VLP

**Fig 2:**
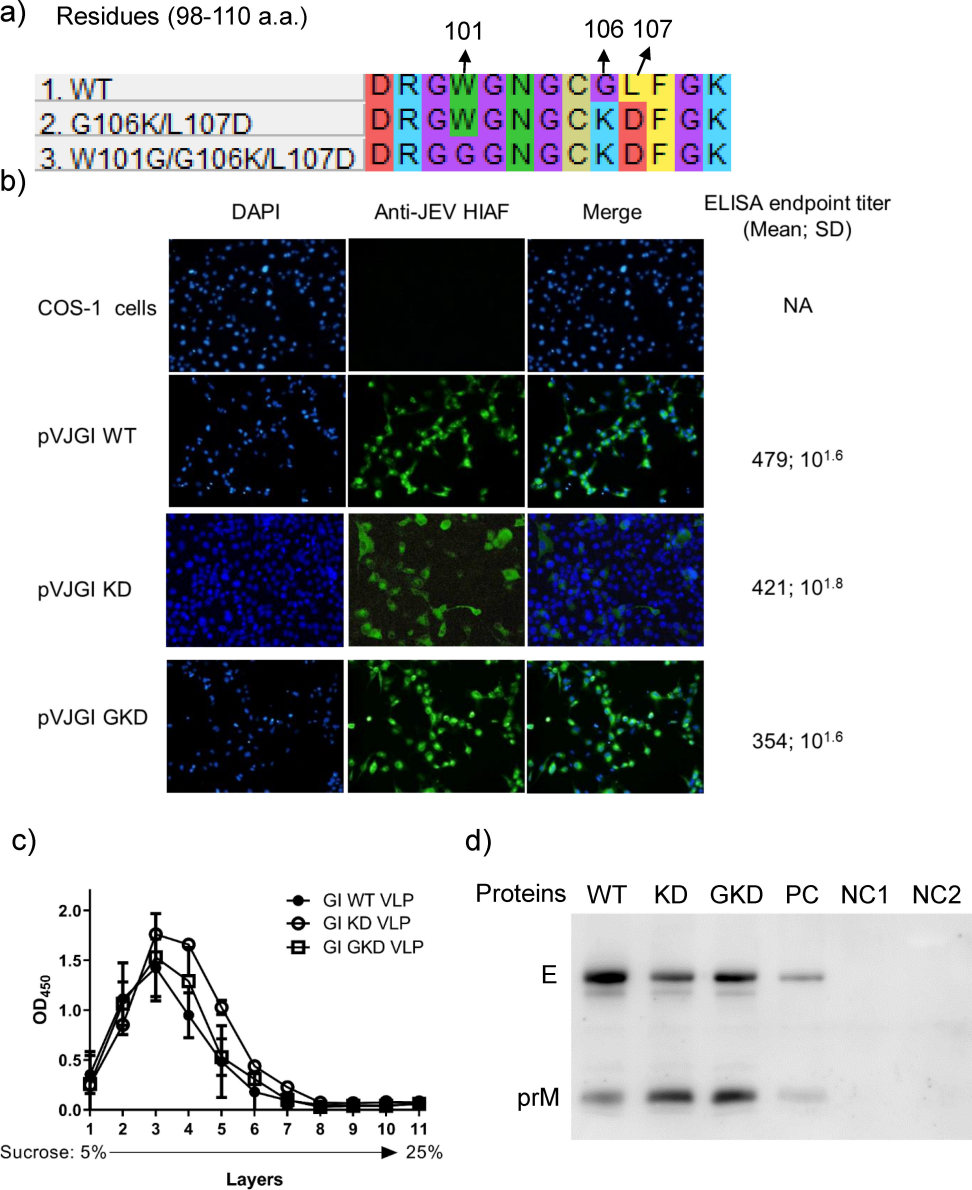
Expression and secretion of GI WT, KD, and GKD VLPs. Expression and secretion of GI WT, KD, and GKD VLPs. (**a**) The fusion loop sequences of pVJGI WT-, pVJGI KD (G106K/L107D), and pVJGI GKD (W101G/G106K/L107D) were presented. (**b**) Mouse anti-JEV hyperimmune ascitic fluid (HIAF) was used to detect the prM/M and E proteins in the pVJGI WT-, pVJGI KD, and pVJGI GKD-transfected COS-1 cells using immunofluorescence assay (IFA). DAPI stained the cellular nuclei. The endpoint titer of the secreted VLPs was measured by Ag-ELISA. The mean and standard deviation were presented in duplicate tests. (**c**) The formation of VLP particles was analyzed by 5% to 25% of sucrose gradient centrifugation. The location of VLPs in the 11 fractions was detected by Ag-ELISA. The error bar was presented as standard deviation. (**d**) The E and prM proteins incorporated on WT, KD, and GKD VLPs (with 2-mercaptoethanol treatment) were separated by SDS page and detected with rabbit anti-JEV HIAF. The supernatants of GI JEV-infected VERO cells and uninfected COS-1 or VERO cells were used as a positive control (PC) and negative control 1 or 2 (NC1 or NC2), respectively.

We then introduced DII_FL_ G106K/L107D (KD) mutations or W101G/G106K/L107D (GKD) mutations onto WT VLP-expressing plasmid (pVJGI WT) to generate pVJGI KD and pVJGI GKD, respectively (Fig. 2a). Compared to pVJGI WT-transfected COS-1 cells, the pVJGI KD- and GKD-transfected cells showed similar expression of intracellular staining patterns for JEV antigens and secreted a comparable yield of GI KD and GKD VLPs (Fig. 2b). We analyzed particle formation of the mutant VLPs by 5% to 25% of the sucrose density gradient. The secreted WT and mutant VLPs were sedimented in comparable layers and condensed at the 3rd fraction in the gradient (Fig. 2c). We further detected E and prM proteins incorporated onto GI WT, KD, and GKD VLPs as well as JEV particles in Western blotting (Fig. 2d). These results suggested that KD and GKD mutations maintained the ability of VLP formation and secretion and preserved its VLP density or structure undifferentiated from WT VLP.

**Fig 3:**
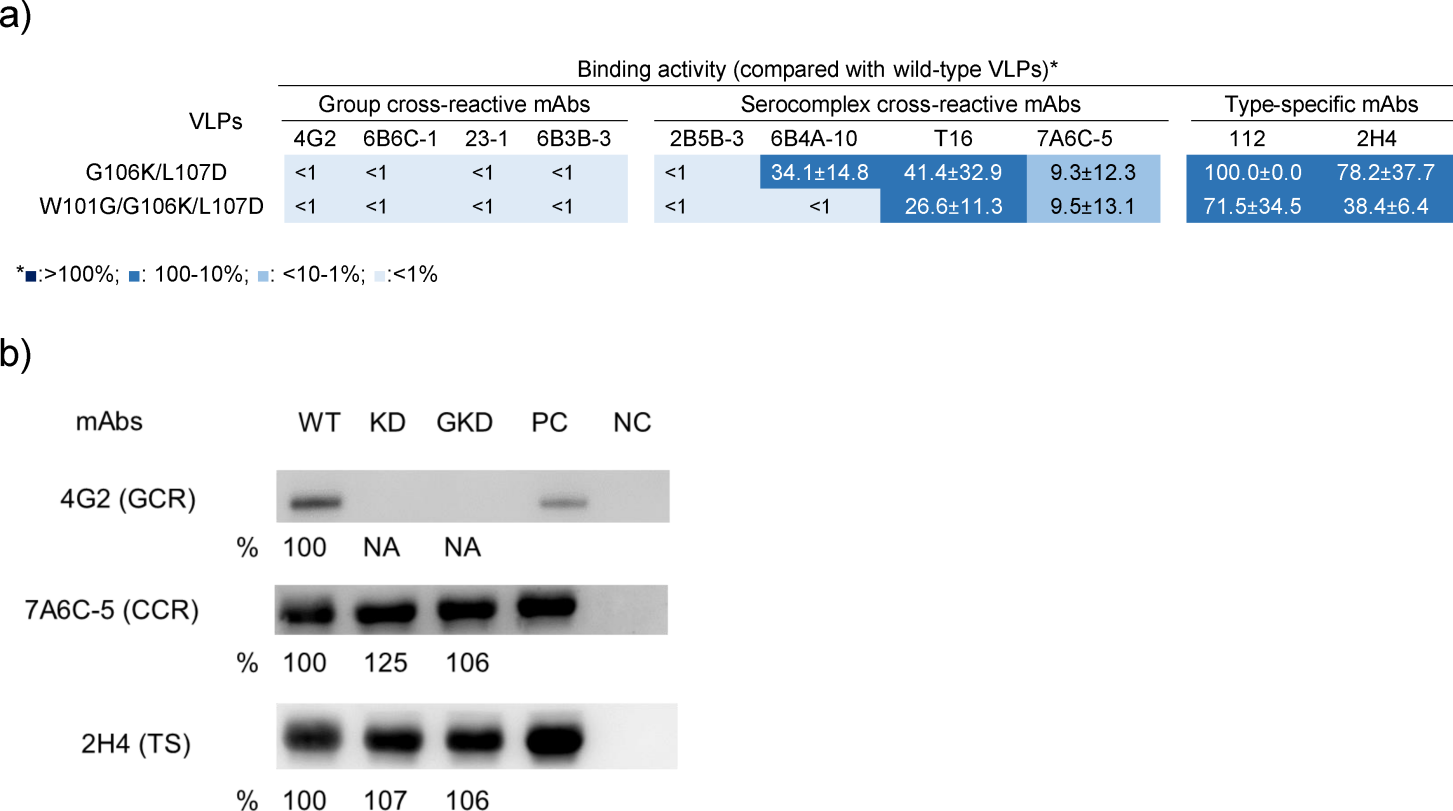
The antigenicity of GI WT, KD, and GKD VLPs. The reactivity of mouse anti-JEV HIAF (MHIAF), group cross-reactive (GCR), complex cross-reactive (CCR), and type-specific (TS) mAbs with KD and GKD VLPs was measured in Ag-ELISA (**a**) and WB analysis (**b**). The mAb binding activity against mutant VLPs was compared to WT VLPs and calculated by [Log ^endpoint titer against (mutant VLP／ WT VLP)^] ×100%. The binding activities were presented as mean ± SD and colored from deep blue to light blue based on their mean percentage of binding (˃100%, 10%–100%, ˂10%–1%, or ˂1%). All experiments were conducted in triplicate. The antigen samples, devoid of 2-mercaptoethanol treatment, were subjected to boiling and subsequently analyzed in a WB assay. The percentage of mAbs binding activity against E proteins was measured using ImageJ software (https://imagej.nih.gov/ij/) and was indicated below the bands. NA stands for not available; WT refers to GI WT VLP; KD refers to GI KD VLP; GKD refers to GI GKD VLP; PC stands for JEV culture media as a positive control; NC stands for cell culture supernatant as a negative control.

We subsequently analyzed the impact of KD and GKD mutations on the antigenic characteristic of respective VLP by the Ag-ELISA and Western blotting using flaviviral mAbs (Fig. 3). As expected, GI KD and GKD VLPs exhibited a similar pattern in reducing binding activity with all group cross-reactive mAbs and two serocomplex cross-reactive mAbs. However, increased binding activity of G106K or L107D VLPs with T16 and 112 mAbs was not detected against KD or GKD VLP (Fig. 3a and b). These results showed that GI KD and GKD VLP altered the group and supercomplex cross-reactive epitopes while preserving the T16 and type-specific epitopes.

### G106K/L107D and W101G/G106K/L107D mutations did not influence the immunogenicity of VLPs

**Fig 4:**
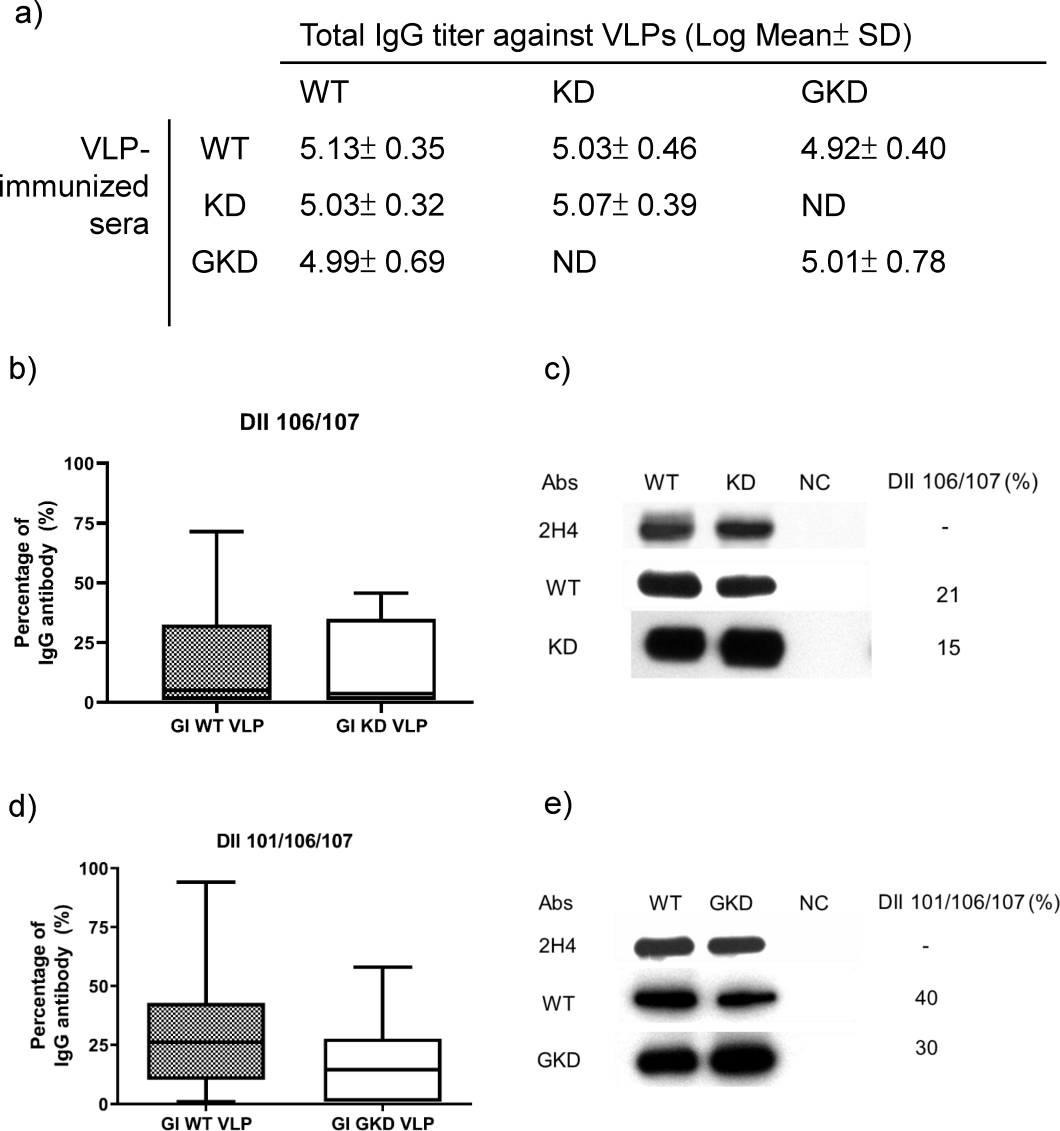
Analysis of antibody responses in GI WT VLP-, KD VLP-, and GKD VLP-immunized mice. (a) Total IgG and DII_FL_ endpoint titers after 3rd immunization were measured by GAC-ELISA using GI WT, KD, and GKD VLPs in GI WT VLP-immunized (*n* = 21), KD VLP-immunized (*n* = 6), and GKD VLP-immunized (*n* = 21) mice. The antibody titers were compared using one-way ANOVA and were measured using GAC-ELISA (a, b, and d) and WB assay (c and d). The percentage of DII_FL_ IgG antibody in GAC-ELISA was calculated by (DII_FL_ IgG titer/Total IgG titer)*100. The box and whisker with 2.5 and 97.5 percentiles were presented. The percentage difference was compared using a two-tailed unpaired *t*-test. In the WB assay, the E proteins of WT, KD, and GKD VLPs (without 2-mercaptoethanol treatment) were detected by 2H4 mAb and the VLP-immunized mice sera. The binding activity of IgG antibody response against the E proteins was measured in GI WT VLP-immunized mouse (WT), KD VLP-immunized mouse (KD), and GKD VLP-immunized mouse (GKD) using ImageJ software (https://imagej.nih.gov/ij/). Percent DII_FL_ IgG antibody response was estimated by the intensity of [(WT – KD or GKD)／WT] × 100, [(KD − WT)／KD] × 100, and [(GKD − WT)/GKD]×100 for GI WT VLP-, KD VLP-, and GKD VLP-immunized mice, respectively. The supernatant of COS-1 cells was used as a negative control (NC) antigen.

The antigenic characterization suggested that GI KD VLP and GKD VLP might induce variant antibody profiles targeting DII_FL_ 106/107 or 101/106/107 residues as compared to WT VLPs. We compared the immunogenicity of these three types of VLPs in immunized mice and found that GI KD and GKD VLP induced similar IgG titers (10^5.07 ± 0.39^ and 10^5.01 ± 0.78^) against the homologous VLP as compared to the WT VLP (10^5.13 ± 0.35^) (*P* ˃ 0.05) (Fig. 4a). We estimated the IgG antibody response targeting DII_FL_ 106/107 and 101/106/107 residues in the immunized mice and found that KD VLP- and GKD VLP-immunized mice elicited a non-significantly different IgG antibody response to DII_FL_ 106/107 and 101/106/107, respectively (*P* ˃ 0.05) (Fig. 4b and d). The Western blotting results also showed a similar staining pattern (Fig. 4c and e). These results demonstrated that GI WT VLP, KD VLP, and GKD VLP as well as their DII_FL_ residues had similar immunogenicity even though the antigenic landscapes were different.

### DII_FL_ G106K/L107D and W101G/G106K/L107D mutations reduced the VLP ability to elicit neutralizing antibodies

**Fig 5:**
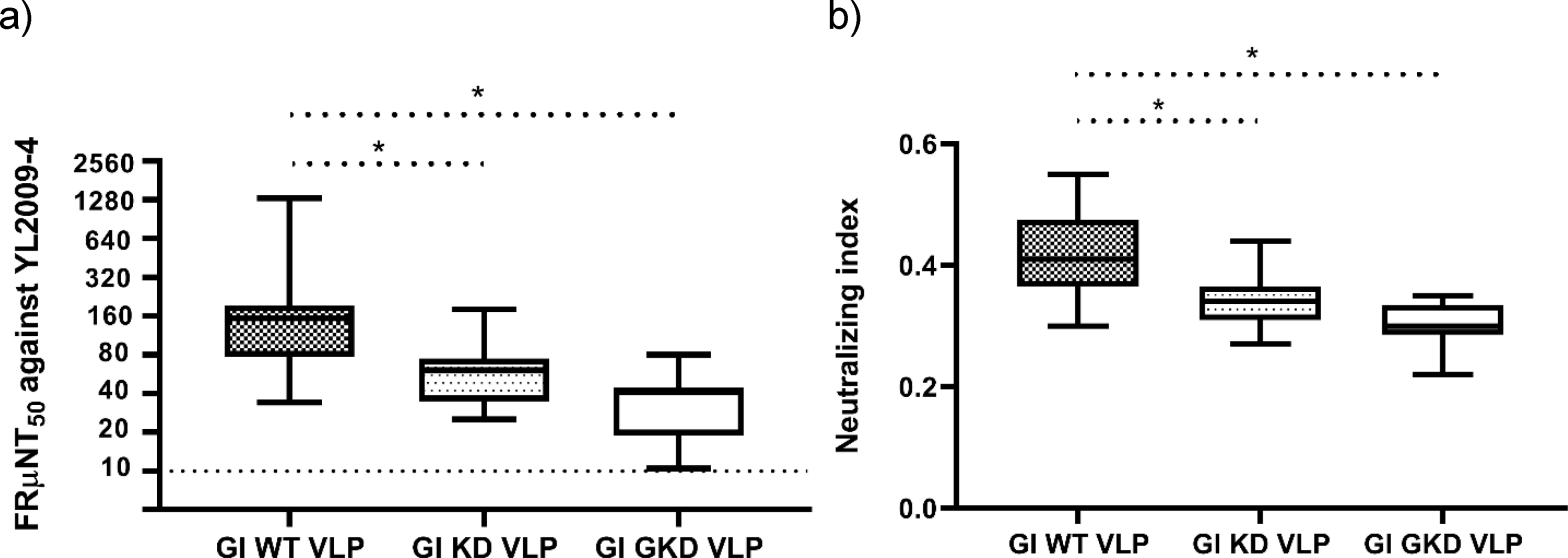
The neutralizing antibody titer against GI YL2009-4 virus and quality of the total IgG antibody response induced by GI WT, KD, and GKD VLPs. (**a**) The neutralizing antibody titers of the mice sera (WT VLP-immunized mice, *n* = 21; KD VLP-immunized mice, *n* = 13; GKD VLP-immunized mice, *n* = 21) were measured by FRμNT_50_ against GI YL2009-4 virus. (**b**) The neutralizing index for each mouse was calculated by the ratio of Log ^FRμNT50 against GI YL2009-4 virus^ to Log ^ELISA IgG endpoint titer^. The box and whisker with 2.5 and 97.5 percentiles were presented. A significant difference (*P* ˂ 0.05) was analyzed using one-way ANOVA with Tukey’s multiple comparisons test and indicated by an asterisk.

The neutralizing antibody titer is considered as an immune correlate of protection against JEV infection but the role of DII_FL_-reactive antibody remains unclear in correlating the protective immunity (14). Reduction of DII_FL_ immunogenicity on immunogens could induce a higher neutralizing antibody response by altering an antibody profile (25, 26). We observed similar immunogenicity of the DII_FL_ residues on the mutant and WT VLPs (Fig. 4). If DII_FL_-reactive antibodies exhibit non- or weak neutralizing activity, KD and GKD VLPs might elicit similar neutralizing antibody response with WT VLPs in mice. Therefore, we measured neutralizing activity of the immunized mice sera to investigate whether KD and GKD mutations influenced the VLP ability to induce neutralizing antibodies (Fig. 5a). Unexpectedly, the KD and GKD VLP-immunized sera showed significantly lower FRμNT_50_ titers (59.6 and 32.4) than the titer of 146.6 for the WT VLP-immunized sera (*P* ˂ 0.05) (Fig. 5a). The quality of antibody response was evaluated by calculating the ratio of FRμNT_50_ titer to the homologous IgG titer for each mouse (Fig. 5b). Overall, GI KD and GKD VLP-immunized mice elicited lower quality of the neutralizing IgG antibody response than WT VLP-immunized mice after third-dose immunization (*P* ˂ 0.05). These results suggested that DII_FL_ 106/107 and 101/106/107 residues were critical for GI VLP to induce potently neutralizing antibodies against GI JEV.

### The anti-DII_FL_ 106/107 and 101/106/107 antibody response provided homologous neutralizing activity

**Fig 6:**
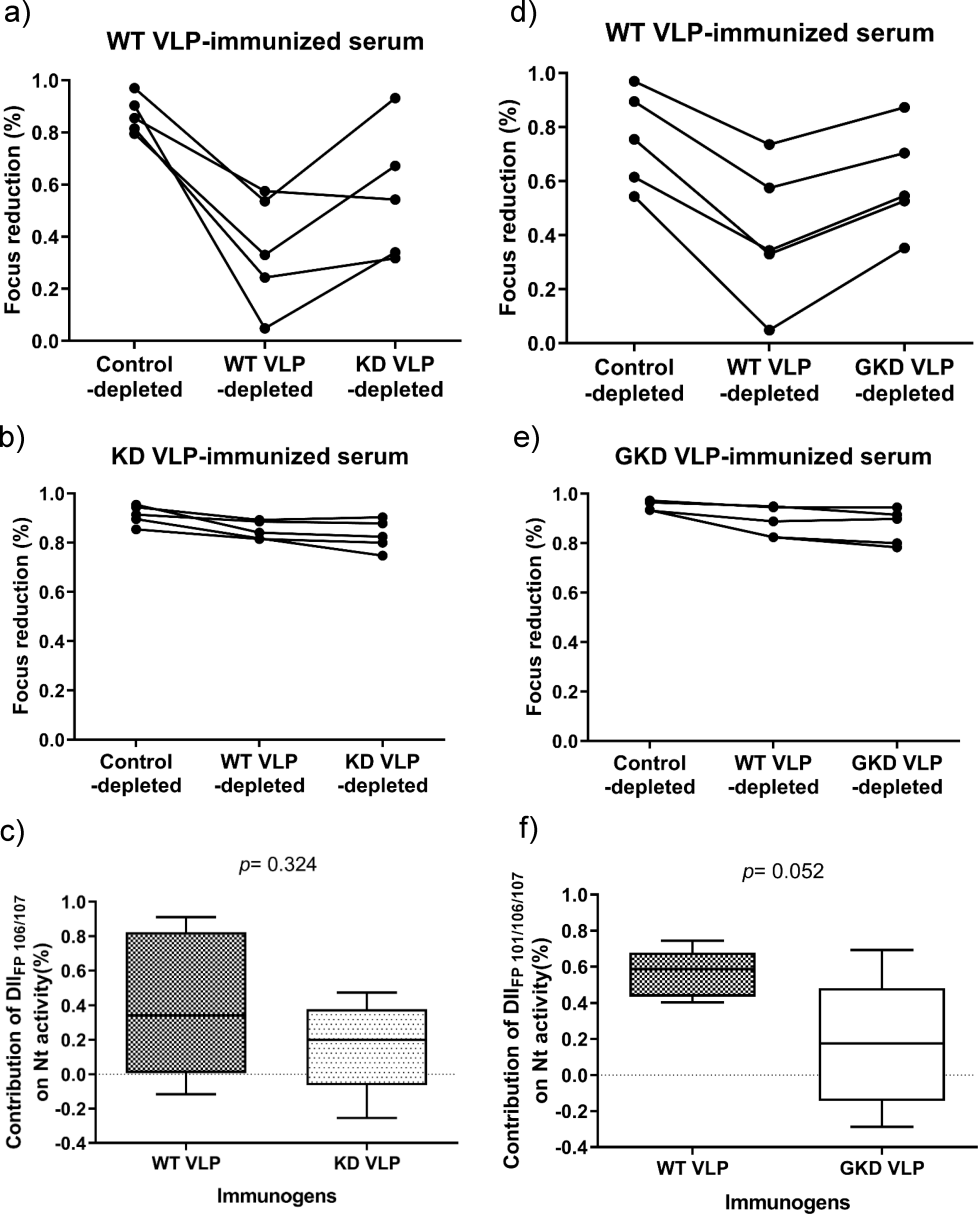
VLP-depletion studies to measuring the neutralizing activity of anti-DII_FL_ antibodies. The serum specimens from immunized mice were incubated with transformed cells expressing WT VLPs (WT VLP-depleted), KD VLPs (KD VLP-depleted), and GKD VLPs (GKD VLP-depleted). Control-depleted serum was obtained by incubating the mice sera with COS-1 cells. After incubation, the neutralizing activity of the unbound serum was measured against GI YL2009-4 virus for WT VLP-immunized (**a and d**), KD VLP-immunized (**b**), and GKD VLP-immunized (**e**) sera. The percentage of focus reduction was calculated for control-depleted, WT VLP-depleted, KD VLP-depleted, and GKD VLP-depleted sera compared to 100% focus reduction for the serum sample without depletion. (**c and f**) The contribution of anti-DII_FL_ 106/107 or 101/106/107 antibodies to neutralizing activity was estimated by the difference in focus reduction between WT VLP-depleted and KD VLP-depleted sera in the WT VLP- and KD VLP-immunized sera (**c**) or between WT VLP-depleted and GKD VLP-depleted sera in the WT VLP- and GKD VLP-immunized sera (**d**), respectively. The difference in contribution to neutralizing activity was compared using a two-tailed unpaired *t*-test.

**Fig 7:**
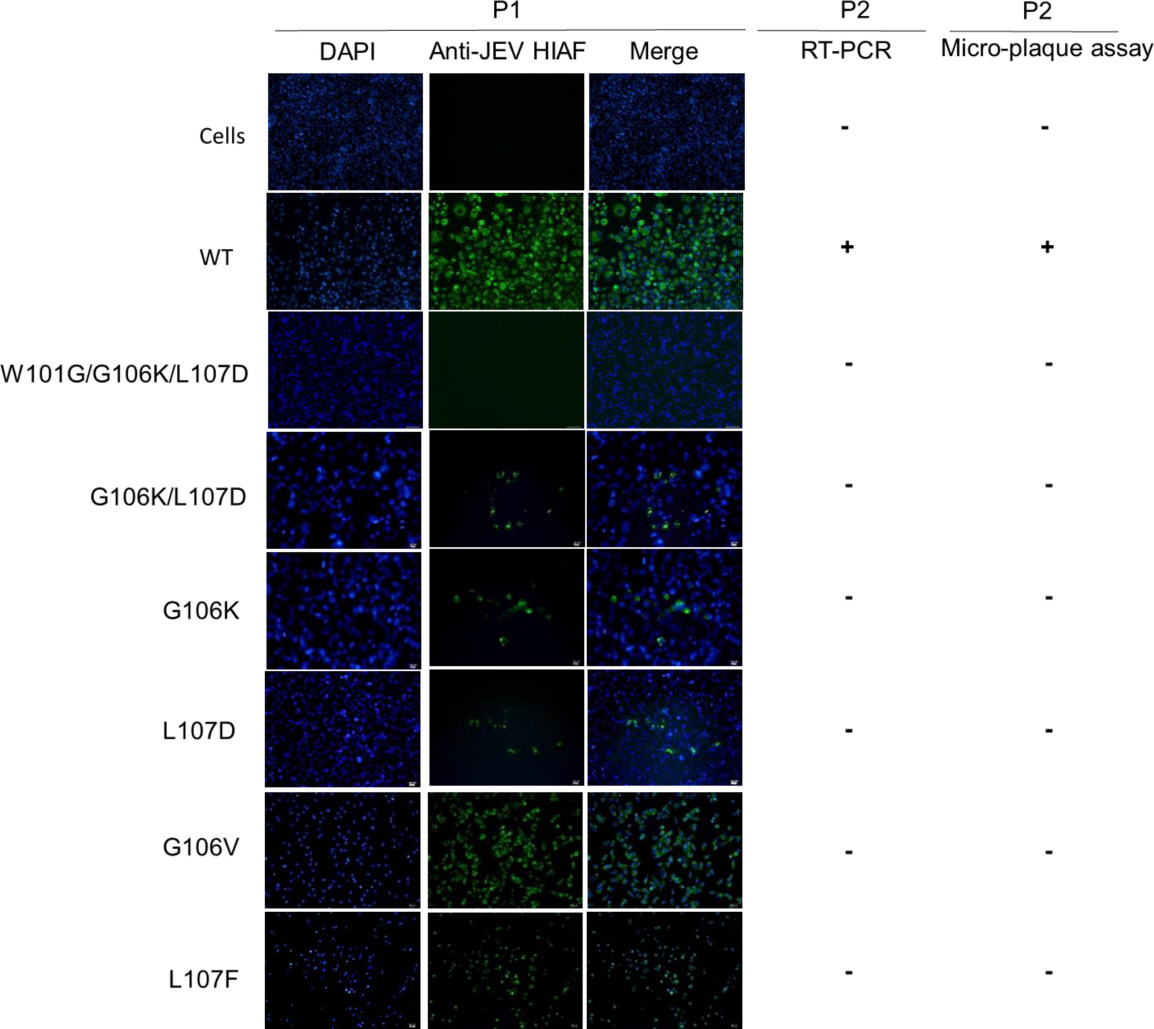
Generation of recombinant GI viruses encoding DII_FL_ mutations. BHK-21 cells were transfected with either pCMV GI WT or pCMV GI mutant clones containing DII_FL_ mutations. The recombinant viruses released from the transfected cells underwent amplification for two passages (**P1 and P2**) in BHK-21 cells. Mouse anti-JEV hyperimmune ascitic fluid (HIAF) was employed to detect prM/M and E proteins in transfected BHK-21 cells using immunofluorescence assay (IFA). Cellular nuclei were stained with DAPI. The viral RNA and infectivity of the recombinant viruses were assessed through RT-PCR and micro-plaque assay, respectively.

We observed the mutant VLPs elicited lower neutralizing antibodies than the WT VLP while inducing similar IgG antibody titers to the homologous VLPs and their DII_FL_ region (Fig. 4). These results implied that W101, G106, and/or L107 in the WT VLPs might elicit antibodies that have a stronger neutralizing activity than the one induced by the mutant VLPs. Thus, we measured the contribution of anti-DII_FL_ antibody response on neutralizing activity by comparing the difference in percent focus reduction of the homologous VLP-depleted sera to the heterologous VLP-depleted sera (Fig. 6). Most WT VLP-immunized sera exhibited lower activity in focus reduction after the antibody depletion with the homologous VLP than with KD or GKD VLPs (Fig. 6a and d). We estimated that the WT 106/107- and 101/106/107-reactive antibodies contributed to 40.0% (95% CI in 2.9% to 77.1%) and 56.2% (95% CI in 44.5% to 67.9%) neutralizing activity in sera from mice immunized with WT VLP, respectively (Fig. 6c and f). By contrast, KD- and GKD-reactive antibodies only contributed 16.5% (95% CI in −7.0% to 39.9%) and 17.0% (95% CI in −10.6% to 48.7%) of the neutralizing activity in sera from mice immunized with KD VLP and GKD VLP, respectively (Fig. 6c and f). The small sample size used in this assay may have resulted in a nonsignificant difference observed in the contribution of DII_FL_-reactive antibodies between sera from WT VLP- and mutant VLP-immunized mice. However, these results did imply that the WT VLPs elicit a portion of neutralizing antibodies binding to WT 106/107 and 101/106/107 residues. To further dissect the neutralizing activity of anti-DII_FL_ antibodies, we constructed infectious clones to generate recombinant GI viruses encoding single, double, and triple mutations at DII_FL_ 101, 106, and 107 residues. Unfortunately, all mutated full-length clones were not viable, and we were unable to recover the infectious and mutant viruses after two passages (Fig. 7).

### The involvement of DII_FL_ 101/106/107 in eliciting cross-protective neutralizing antibodies

**Fig 8:**
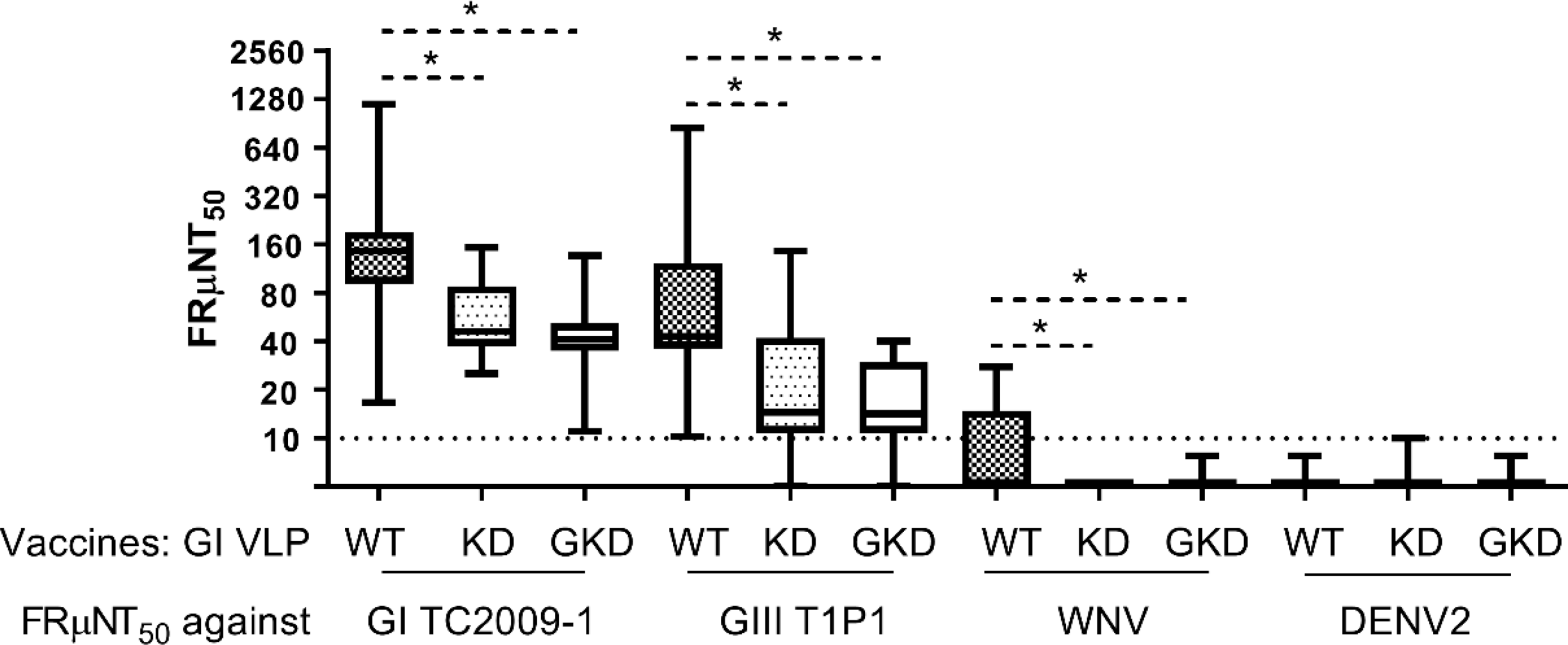
Cross-neutralizing activity against GI JEV, GIII JEV, WNV, and DENV2 in GI WT VLP-, KD VLP-, and GKD VLP-immunized mice. The neutralizing activity of the mice sera (WT VLP-immunized mice, *n* = 21; KD VLP-immunized mice, *n* = 13; GKD VLP-immunized mice, *n* = 21) was measured by FRμNT_50_ against GI TC2009-1 virus, GIII T1P1 virus, WNV, or DENV2. The box and whisker with 2.5 and 97.5 percentiles were presented. The dashed line indicates the detection limit. The FRμNT_50_ titers of GI WT VLP-, KD VLP-, and GKD VLP-immunized mice were compared using one-way ANOVA with Tukey’s multiple comparisons test. A significant difference (*P* ˂ 0.05) was indicated by an asterisk.

The KD and GKD mutations reduced the ability of VLP to induce neutralizing antibodies binding to WT 101/106/107 residues (Fig. 5 and 6). Since the amino acid sequences of DII_FL_ 101/106/107 residues are conserved across flaviviruses (45), the DII_FL_ mutations might affect the VLP-induced cross-protective neutralizing antibodies against all or some flaviviruses. We measured the FRμNT_50_ titers against GI virus, GIII virus, WNV, and DENV2 for sera from mice immunized with KD and GKD VLPs (Fig. 5a and 8). The sera from mice immunized with GI KD VLPs and GI GKD VLPs had a 2.6- and 3.5-, 2.7- and 3.4-, 1.6- and 1.6-fold reduction in neutralizing activity against GI TC2009-1 virus, GIII T1P1 virus, and WNV, respectively, compared to sera from WT VLP-immunized mice. However, only 42.9% (9/21) of sera from mice immunized with WT VLPs demonstrated low neutralizing titers against WNV. None of the sera from immunized mice could neutralize DENV2, except for one KD VLP-immunized mouse, which exhibited a FRμNT_50_ titer of 10. This result indicated that KD and GKD VLPs induced lower JEV type-specific or/and JE serocomplex cross-reactive, neutralizing antibodies. A greater reduction in averaged FRμNT_50_ titers against GI TC2009-1 virus and GIII T1P1 virus than WNV suggested KD and GKD VLPs might induce lower type-specific neutralizing antibodies. These results demonstrate that DII_FL_ 101/106/107 residues play a role in eliciting neutralizing antibodies, which might be JE serocomplex cross-reactive or/and JEV type specific.

### GI DII_FL_-mutant VLPs reduced the ability to elicit protective immunity against JEV infection

**Fig 9:**
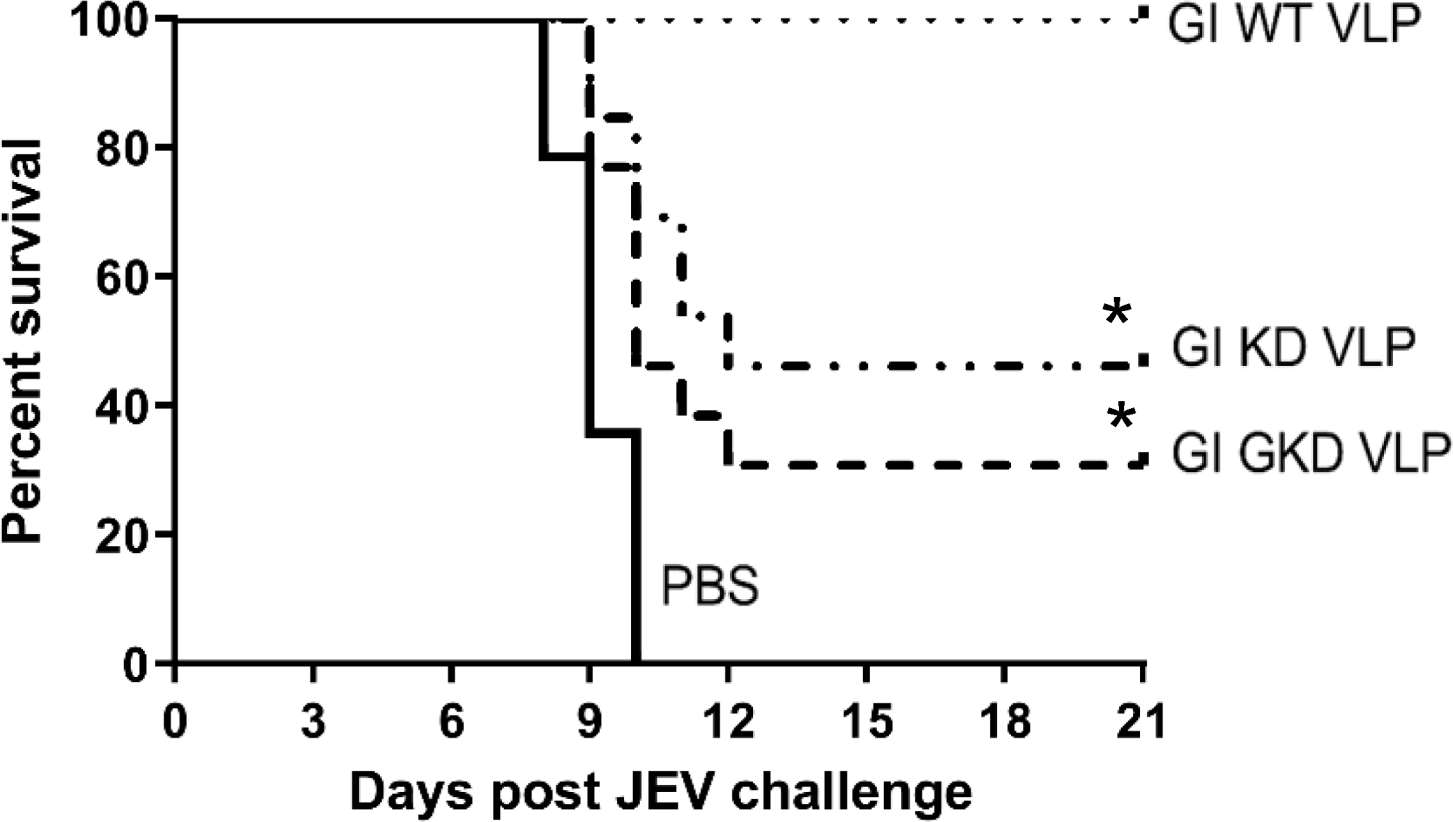
The survival rate of GI WT VLP-, KD VLP-, and GKD VLP-immunized mice after challenge with GI YL2009-4 virus. The survival curve of PBS- (*n* = 14), GI WT VLP- (*n* = 12), GI KD VLP- (*n* = 13), and GI GKD VLP- (*n* = 13) immunized mice were compared using the Log-rank test. A significant difference (*P* ˂ 0.05) was indicated by an asterisk compared to GI WT VLP-immunized mice.

We observed that KD and GKD VLPs induced lower neutralizing antibody titers (Fig. 5) and that these titers were positively correlated with JEV vaccine potency (14). The VLP-immunized mice were challenged with GI YL2009-4 virus after the third dose of immunization. All the phosphate-buffered saline (PBS)-immunized mice were dead at 10 days post-challenge. All the GI WT VLP-immunized mice (12/12) survived for 21 days, while only 46% (6/13) and 31% (4/13) of the KD and GKD VLP-immunized mice survived, respectively, with an average survival time of 12 and 10 days (Fig. 9). These results demonstrated that KD and GKD mutations significantly reduced the ability of GI VLPs to protect mice from lethal JEV infection (*P* ˂0.05). The result was consistent with our observation that the neutralizing activity of mutant VLP-immunized mouse sera was 2.5-fold to 4.5-fold lower than that of WT VLP-immunized mouse sera (Fig. 5a) and suggested that JEV WT-DII_FL_ 101/106/107 residues binding antibodies might play an important role in protective immunity against JEV infection.

### Reduced induction of neutralizing antibodies results in a lower survival rate in GI DII_FL_-mutant VLP-immunized mice

We further investigated whether the reduced production of neutralizing antibody response contributed to the decreased survival rate in the KD VLP- and GKD VLP-immunized mice. We believe using the same intracranial route of the JEV challenge in the passive transfer experiment would provide a more comparable context to Fig. 9. Naive mice received pooled sera from PBS-immunized-, GI WT VLP-, GI KD VLP-, or GI GKD VLP-immunized mice and were subsequently challenged with GI JEV *via* the intracranial route (Table 1). We observed an average threefold to fourfold decline in neutralizing titers of injected mouse serum after circulating from the peritoneal cavity to the blood at 16 hours post-inoculation. All mice that received sera from PBS-immunized mice succumbed to the lethal virus challenge. Mice that received sera from WT VLP-immunized mice exhibited a GMT of 31.1 for FRμNT_50_ against GI YL2009-4 virus before the virus challenge, with 46.7% (7/15) of them surviving after the challenge. By contrast, those that received sera from KD VLP-immunized mice and GKD VLP-immunized mice displayed GMTs of 10.5 and 5.9, respectively, and only 6.7% (1/15) and 0% (0/15) of them survived the challenge. This result suggests that DII_FL_ KD and GKD mutations impair the ability of GI VLPs to induce neutralizing antibodies, resulting in a significantly lower protective rate (*P* ˂ 0.05) against homologous JEV infection in VLP-immunized mice.

**TABLE 1:** Passive protection of mice against lethal JEV challenge following pre-treatment with sera from GI WT-, KD-, or GKD-VLP-immunized mice

Groups^*a*^	FRμNT_50_ of input sera^*b*^	FRμNT_50_ of sera before the virus challenge^*c*^	Survival rate (%)^*d*^	Average time to death (days ± SD)
PBS	<5	<5	0 (0/15)^*e*^	8.2 ± 1.1
GI WT VLP	112.2 ± 4.7	31.1 ± 5.3	46.7 (7/15)	8.4 ± 1.3
GI KD VLP	44.6 ± 8.3	10.5 ± 0.6	6.7 (1/15)^*e*^	8.2 ± 0.7
GI GKD VLP	24.1 ± 8.1	5.9 ± 2.9	0 (0/15)^*e*^	7.7 ± 0.7

^*a*^ Fifteen naïve female BALB/c mice per group (PBS, GI WT VLP, GI KD VLP, or GI GKD VLP) were intraperitoneally injected with pooled sera collected from PBS-immunized mice, GI WT VLP-immunized mice, GI KD VLP-immunized mice, or GI GKD VLP-immunized mice after the third dose of immunization.

^*b*^ The FRμNT_50_ titers of pooled sera collected from PBS-immunized mice, GI WT VLP-immunized mice, GI KD VLP-immunized mice, or GI GKD VLP-immunized mice were measured against the GI YL2009-4 strain. FRμNT_50_ titers were presented as geometric mean titers (GMT) ± standard deviation (SD).

^*c*^ Serum specimens were collected from mice at 16 hours post-administration of the pooled sera and subsequently measured for FRμNT_50_ against the GI YL2009-4 strain. FRμNT_50_ titers were presented as GMTs ± SD.

^*d*^ The mice were intracranially challenged with a 100-fold LD_50_ dose of GI YL2009-4 virus at 18 hours post-administration of the pooled sera. Survival of the mice was recorded until 21 days post-challenge. Differences in mouse survival curves were compared to the GI WT VLP group using the Log-rank test.

^*e*^ Statistical significance was considered as *P* < 0.05.

## DISCUSSION

This study elucidates the role of DII_FL_ in eliciting protective immunity against JE serocomplex viruses by comparing the immunogenicity and protective potency of GI DII_FL_-mutated VLPs with GI WT VLPs in mice. We observed that the mutant DII_FL_ reduced reactivity with the group and JE serocomplex cross-reactive antibodies but maintained similar immunogenicity compared to the WT VLP. The DII_FL_-mutated VLPs elicited less neutralizing antibodies against JEV and WNV compared to WT VLPs. Mice immunized with DII_FL_-mutated VLPs had a lower survival rate than those immunized with WT VLPs after lethal JEV challenge. That lower viral neutralizing activity of sera collected from DII_FL_-mutated VLP-immunized mice resulted in reduced protective efficacy against homologous virus infection. These results demonstrated and supported that JEV DII_FL_ 101/106/107 residues were involved in inducing cross-neutralizing antibodies and protective immunity against virus infection. Our findings, along with previous studies, emphasized the pivotal role of neutralizing antibodies in protecting mice against lethal JEV infection (14, 46). Nonetheless, we cannot rule out the potential impact of the DII_FL_ mutation on the induction of T-cell responses.

A single DII_FL_ mutation, G104H, significantly reduced the GI VLP yields in this study. This reduction was also observed for DENV-2 and GIII JEV VLPs (38, 42). The structural location of G104 residue on an immature or mature virion suggests that G104H mutation might interfere with the interaction between DII_FL_ and prM or glycan during VLP assembly or/and secretion (47) (Fig. 1). Introducing a mutation at W101 may potentially disrupt the formation of the E dimer due to its structural location. However, our findings, along with previous studies, have demonstrated the successful generation of flavivirus VLPs encoding W101G or W101A mutations, which retain the antigenic epitopes recognized by type-specific or DIII-reactive mAbs (43, 44). The fusion loop epitope (FLE)-specific mAbs, which recognized the W101 residue alone or with other DII_FL_ residues, showed low neutralizing activity against flavivirus (42, 48–50). The newly identified EDE-specific mAbs also recognized the DII_FL_ 101 residue had potent cross-neutralizing activity against the DENV serocomplex or DENV-ZIKV super serocomplex group (48, 51). Some DII_FL_-reactive JE serocomplex mAbs that recognized residue(s) on the nearby DIII of the other E monomer might have higher neutralizing activity than the other serocomplex mAbs targeting DII_FL_ alone (37, 38, 43, 50, 52). JEV type-specific mAbs recognizing DII_FL_ remained unidentified from the virus-infected or vaccinated mice and humans (35). While observing a drastic reduction in the neutralizing activity against JEV than WNV between WT and DII_FL_-mutated VLP-immunized sera, we speculate that the impact on induction of serocomplex cross-reactive antibodies might have higher neutralizing activity against JEV than WNV. We were unable to exclude the potential impact of DII_FL_ mutations on the induction of type-specific and neutralizing antibodies although two type-specific mAbs used here remained binding activity with the DII_FL_-mutated VLPs. A human-derived mAb-targeted DII_FL_ 101, 104, and 106 residues could prevent mice from WNV and DENV infections (53, 54). E53 mAb, targeted DII_FL_ 106 and 107 but not 101 residue, could still provide partial protection against WNV infection (50). These studies suggested that DII_FL_ 101, 106, and 107 residues might be involved in inducing non-neutralizing as well as cross-neutralizing antibodies.

Here, we demonstrated DII_FL_ 106/107 residues were involved in inducing neutralizing antibodies against JEV and these two residues together with the 101 residue were involved in eliciting neutralizing antibodies against JEV and WNV. However, a recent study showed that JEV GIII VLP-expressing plasmids encoding single or double DII_FL_ G106V and L107F mutations performed comparably to the wild-type plasmid in eliciting neutralizing antibodies against JEV (39). This discrepancy could be due to the use of different amino acids at the DII_FL_ 106 and 107 residues, the method to measure neutralizing antibody titers, or the type of immunogens (VLP vs DNA) used in the study (39). By contrast, DENV2 VLP-expressing plasmids encoding DII_FL_ G106R/L107D mutations elicited comparable neutralizing antibodies to the wild-type plasmid by reducing DII_FL_ immunogenicity and manipulating antibody profile (30). This result was consistent with evidence that DENV DII_FL_ G106/L107 residues were not the part of highly neutralizing EDE epitope (48). Our study showed that JEV DII_FL_ maintained its immunogenicity after introducing 101/106/107 mutations into the GI VLP, suggesting that DII_FL_ 106 and 107 residues may play a different role in inducing antibodies and protective immunity against JE serocomplex virus compared to DENV serocomplex virus. However, DENV1 and ZIKV immunogens encoding DII_FL_ 106/107 or 101/107 mutations elicited a lower neutralizing antibody response than its WT immunogens (29, 55). A single DII_FL_ F108A mutation altered particle maturity for ZIKV VLP and reduced its ability to induce protective immunity (32). However, these studies did not address the independent role of DENV1 and ZIKV DII_FL_ in eliciting protective immunity.

The fusion loop is conserved in flavivirus and antibodies with footprint-covered fusion loop residues dominate the immune response. DENV infection induces most antibodies targeting the DII_FL_ 101 residue, followed by the 106/107/108 residues in humans (56, 57). By contrast, JEV- or WNV-infected humans produced less antibody responses targeting DII_FL_ 106 and 107 residues (38). Our study also demonstrated low immunogenicity of DII_FL_ on GI WT VLP, KD VLP, and GKD VLP. DENV or ZIKV infection induced non-neutralizing and anti-DII_FL_ antibodies associated with developing ADE-related severe dengue upon subsequent infection (11, 58, 59). DENV and ZIKV immunogens with DII_FL_ mutations reduced the occurrence of ADE *in vitro* and *ex vivo* by reducing DII_FL_ immunogenicity and/or the binding activity of anti-DII_FL_ antibodies to the virion (29–32, 55). The ADE of JEV and WNV infection was observed *in vitro* assay only; and this phenomenon requires the support of epidemiological evidence (4, 13, 60–62). Further studies are required to evaluate the benefit of using DII_FL_-mutated vaccine candidates of JE serocomplex virus to reduce the risk of ADE for DENV or WNV infection after JEV vaccination.

Our results and other studies suggest a different role of DII_FL_ in DENV/ZIKV and JEV to elicit protective and/or pathogenic immunities. DII_FL_-mutated immunogens could be a superior next-generation vaccine candidate against DENV and ZIKV but not for JEV in terms of vaccine efficacy and safety, particularly since DENV vaccine clinical trials showed the potential to enhance disease severity among seronegative children after vaccination (8, 63). In addition to E antibody responses, the elicitation of T-cell responses and/or NS1-induced antibody responses proved to be crucial for flavivirus vaccines (64–66). The concurrent activation of CD4^+^ and CD8^+^ T-cell responses in mice demonstrated enhanced efficacy against JEV infection compared to individual CD4^+^ or CD8^+^ T-cell responses alone (67, 68). Mice exclusively receiving vaccine-induced CD4^+^ T cells exhibited a higher survival rate than those receiving transferred CD8^+^ T cells following a lethal JEV challenge (68). A more robust interferon-gamma (IFN-γ)-dominated CD4^+^ T-cell response was correlated with improved recovery from JE clinical outcomes, with the potential capacity to recognize non-DII_FL_ regions of the E proteins (69). Both CD4^+^ and CD8^+^ T-cell responses were essential for controlling and recovering from WNV infection in mice (70–72), and virus-specific CD8^+^ T cells were more abundant than CD4^+^ T cells in WNV-infected humans (73). CD4^+^ and CD8^+^ T cells induced by DENV and ZIKV contributed to protection against viral infection (10, 74–77), with a particular emphasis on the critical role of CD8^+^ T-cell responses against heterologous serotype infections or flavivirus infections (10, 76–78). In contrast to WNV, ZIKV, and DENV, limited evidence supports the pivotal role of CD8^+^ T-cell responses against JEV infections. Further studies are required to determine whether DII_FL_ mutations have an impact on vaccine-induced T-cell responses.

In conclusion, JEV vaccines should preserve the intact of DII_FL_ to elicit neutralizing antibodies and provide protective immunity against JEV or JE serocomplex virus infections. The DII_FL_ L107F mutation has been used to develop attenuated JE serocomplex virus vaccines based on the commonly used GIII vaccine strain (79–82). Further research is needed to clarify the independent role of DII_FL_ W101, G106, or L107 in inducing protective B cells and T-cell immunity against JE serocomplex virus infection (12).

## MATERIALS AND METHODS

### Cells, viruses, and flaviviral antibodies

We maintained COS-1 cells and Vero cells (kindly gifted by Dr. GJ Chang, retired from US Centers for Disease Control and Prevention, Fort Collins, CO) in Dulbecco’s modified Eagle’s minimal essential medium (DMEM, Gibco) supplemented with 10% and 5% heat-inactivated fetal bovine serum (FBS, Gibco), respectively, in a 37℃ incubator with 5% CO_2_. BHK-21 cells were cultured in Minimum Essential Medium (MEM, Gibco) with 10% heat-inactivated FBS in a 37℃ incubator with 5% CO_2_. C6/36 cells were cultured in DMEM media with 5% FBS and incubated at 28℃ with 5% CO_2_. JEV genotype I (GI) subcluster I TC2009-1 strain, GI subcluster II YL2009-4 strain (83), genotype III (GIII) T1P1 strain (84), dengue virus serotype II (DENV2) 16681 strain, and West Nile virus (WNV) NY99 strain (85) were amplified in C6/36 cells and collected from the supernatant at 3–5 days post-infection. This study used a panel of flavivirus-specific monoclonal antibodies (mAbs), including flavivirus group cross-reactive (4G2, 6B6C-1, 23–1, and 6B3B-3), supercomplex cross-reactive (2B5B-3), JE complex cross-reactive (6B4A-10, T16, and 7A6C-5), and JEV type-specific (112 and 2H4) mAbs. Mouse anti-JEV hyperimmune ascitic fluid (MHIAF) and rabbit anti-JEV polyclones were kindly provided by Dr. GJ Chang.

### Construction of pVJGI WT and mutant VLP plasmids

Our previous study used pVJGI WT VLP plasmid to produce genotype I (GI) WT VLPs (41). In this study, we introduced W101G, G104H, G106K, L107D, F108A, G106K/L107D, or W101G/G106K/L107D mutations into the fusion loop on the pVJGI WT VLP plasmid to generate pVJGI W101G, G104H, G106K, L107D, F108A, KD, or GKD VLP plasmids by PCR reaction (KOD Hot Start DNA Polymerase Merck, United States) using the primers listed in Table 2. The PCR product was treated with DpnI (New England Biolabs, United States) and then transferred into DH5α-competent cells. We extracted pVJGI W101G, G104H, G106K, L107K, F108A, KD, or GKD VLP plasmids from the recovered colony and confirmed the mutations by sequencing (Fig. 1c and 2a).

**TABLE 2:** Primers used for construction of the mutant VLP-expressing plasmids and infectious clones

Mutations	Nt substitutions	Primers (5′−3′)
W101G	TGG-GGC	TCCACATCCATTTCCgccTCCGCGATCAGTAAAGC
G104H	GGA-CAT	CCTTTCCCGAAAAGTCCACAatgATTTCCCCATCCGCG
G106K	GGA-AAA	CCTTTCCCGAAAAGtttACATCCATTTCCCCATCCGCG
G106V	GGA-GTC	CAATGCTTCCTTTCCCGAAAAGgacACATCCATTTCCCCATCCG
L107D	CTT-GAC	CCTTTCCCGAAgtcTCCACATCCATTTCCCCATCCGCG
L107F	CTT-TTC	CAATGCTTCCTTTCCCGAAgaaTCCACATCCATTTCCCCATCCG
F108A	TTC-GCA	CAATGCTTCCTTTCCCtgcAAGTCCACATCCATTTCC
G106K/ L107D	GGA-AAA CTT-GAC	CCTTTCCCGAAgtctttACATCCATTTCCCCATCCGCG
W101G/ G106K/ L107D	TGG-GGC GGA-AAA CTT-GAC	CCTTTCCCGAAgtctttACATCCATTTCCgccTCCGCG

### Expression and purification of VLPs

We transferred 30 µg of pVJGI WT, W101G, G104H, G106K, L107D, F108A, KD, or GKD VLP plasmids into 10^7^ COS-1 cells in 0.4-cm-electrode-gap cuvettes by electroporation at 250 V/ 975 µF with a Bio-Rad Gene Pulser II (Bio-Rad Laboratories, Hercules, CA). The transformed cells were recovered in a 37℃ incubator overnight and then transferred to a 28℃ incubator to enhance the secretion of VLPs in serum-free medium (SFM4MegaVirTM, HyCloneTM) with the addition of 1× cholesterol (Gibco) (38). After 3- to 5-day incubation, WT and mutant VLPs were collected from the supernatant of the transfected COS-1 cells. We concentrated the VLPs by Amicon Ultra-15 Centrifugal Filter Unit (100,000 molecular weight cut-off, Merck, United States) and then proceeded to 20% sucrose density centrifugation with 19,000 rpm at 4℃ for 16 hours. The VLP pellets were suspended in 1× TNE buffer at 4℃ overnight. We layered 25% to 5% of sucrose into an ultracentrifuge tube (Beckman Coulter Inc., CA) from bottom to top and incubated it at 4℃ overnight to form the density gradient. Then, the concentrated VLPs were analyzed by 5% to 25% of sucrose density gradient centrifugation with 25,000 rpm at 4℃ for 3 hours. One milliliter of fractionating layers was collected from top to bottom. We detected VLP antigens in the fractionating layers by antigen-capture enzyme-linked immunosorbent assay (Ag-capture ELISA). The peak OD_450_ values of GI WT, KD, or GKD VLPs in the gradient were purified using Amicon® Ultra-4 Centrifugal Filter Unit (100,000 molecular weight cut-off, Merck, United States) and recovered in 1× PBS. The purified VLPs were used to immunize mice.

### Indirect immunofluorescence assay

We fixed transfected COS-1 cells with 4% paraformaldehyde-PBS and detected VLP antigens in the cells using mouse anti-JEV HIAF. FITC-conjugated goat anti-mouse IgG antibodies (KPL, Gaithersburg, MD) bound to mouse anti-VLP antibodies and DAPI (Invitrogen) stained cell nucleus. The positive signal for JEV antigens and cell nuclei was observed and captured by OLYMPUS CKX41.

### Antigen-capture enzyme-linked immunosorbent assay

We coated 96-well plates with rabbit anti-JEV polyclonal antibodies at 37°C for 1 hour and blocked them with StartBlock blocking buffer (Pierce, Rockford, Ill.). Next, we added VLPs or negative COS-1 cell antigens into the wells and incubated them at 4°C overnight. The captured antigens were recognized by serially diluted mouse anti-JEV HIAF or mAbs at 37°C for 1 hour. The bound mouse anti-JEV antibodies were detected by peroxidase-conjugated goat anti-mouse IgG (H + L) (Jackson ImmunoResearch, West Grove, PA) at 37°C for 1 hour. Unbound antibodies were discarded by washing with 1× PBST. Finally, we added TMB substrate (Neogen Corp., Lexington, KY) to the 96 wells, which reacted with peroxidase for 10 minutes. We then added 2N H_2_SO_4_ to stop the coloring reaction and measured and recorded the values of OD_450_. The ELISA endpoint titer of the VLP antigens was presented as the reciprocal dilution reaching the OD_450_ ratio of the sample to negative antigens (P/N ratio) equal to 2 in GraphPad version 5.01. The mAb-binding activity against mutant VLPs was compared to WT VLPs and calculated by [Log ^endpoint titer against (mutant VLP／ WT VLP)^] ×100%.

### SDS-PAGE and Western blot

We mixed GI YL2009-4 virus, GI WT VLP, KD VLP, GKD VLP, or COS-1 cell supernatants with the 5× sodium dodecyl sulfate-polyacrylamide (SDS) non-reducing sample buffer (315 mM Tris, pH = 6.8, 50% glycerol, 5% SDS, 0.025% bromophenol blue) or with an addition of 2-Mercaptoethanol and denatured them in boiled water for 10 minutes. The boiled mixtures were electrophoretically analyzed by 10% SDS-PAGE at 80V for 30 minutes and 120V for 70 minutes. The separated proteins on SDS gels were transferred to nitrocellulose membranes at 200V for 3 hours. The proteins on the membranes blocked with 5% skim milk-PBST were detected with rabbit anti-JEV polyclones, mAbs (4G2, 7A6C-5, and 2H4), and serum specimens collected from GI WT VLP, KD, and GKD VLPs-immunized mice at 37℃ for 1 hour. The membranes were washed with 1× PBST and then incubated with peroxidase-conjugated goat anti-rabbit or anti-mouse IgG (H + L) (Jackson ImmunoResearch, West Grove, PA) at 37℃ for 1 hour. Antibody-reactive proteins were visualized with the LumiGOLD™ ECL Western Blotting Detection Kit (SignaGen Laboratories, Gaithersburg, MD). We estimated the percent DII_FL_ IgG antibody response by the intensity of [(WT – KD or GKD)／WT]*100, [(KD − WT)／KD]*100, and [(GKD − WT)／GKD]*100 for GI WT VLP-, KD VLP-, and GKD VLP-immunized mice by Image J 1.44 version, respectively.

### Mouse experiments

We immunized female BALB/c mice with three doses of PBS (14 mice), 40 ng of GI WT VLP (21 mice), GI KD VLP (13 mice), or GI GKD VLP (21 mice) mixed with Freund’s incomplete adjuvant (SIGMA-ALDRICH) according to the previous study (41). The mice received 1st dose, 2nd dose, and 3rd dose at 4, 8, and 12 weeks old. We collected mouse blood 4 weeks after the third dose and subsequently centrifuged it at 3,000 rpm for 15 minutes to obtain serum specimens. Six weeks after the final booster, we intracranially injected the immunized mice with 100-fold LD_50_ of homologous JEV GI YL2009-4 strain and recorded the survival rates of mice twice daily until 21 days post-challenge.

In the passive protection experiment, the mouse serum specimens collected after the third dose were pooled and subsequently inactivated at 56℃ for 30 minutes. Subsequently, 60 female BALB/c mice were intraperitoneally injected with 0.5 mL of the pooled mouse sera obtained from PBS-, GI WT VLP-, GI KD VLP-, or GI GKD VLP-immunized mice. At 16 hours post-serum transfer, mouse sera were collected and assayed for the neutralizing activity. Previous research estimates that 0.1% to 1% of circulating antibodies can traverse the intact blood-brain barrier (BBB) and enter the murine brain (86, 87). Intracranial injection of JEV could disrupt the BBB, leading to heightened levels of passively transferred serum IgG in murine brains, as observed in other neurotropic flaviviruses such as WNV (88, 89). Therefore, at 18 hours post-serum transfer, each mouse received an intracranial injection of a 100-fold LD_50_ dose of the JEV GI YL2009-4 strain. Survival rates of the mice were recorded twice daily until 21 days post-challenge.

### Mouse IgG antibodies-capture enzyme-linked immunosorbent assay

To detect IgG antibody responses in the immunized mice, we used a mouse IgG antibodies-capture enzyme-linked immunosorbent assay (GAC-ELISA) as described previously (30, 57, 90). Briefly, we coated 96-well immunoplates (Sigma-Aldrich, St. Louis, MO) with goat anti-mouse IgG (H + L) (KPL, Gaithersburg, MD) at 37°C for 1 hour and blocked them with StartBlock blocking buffer (Pierce, Rockford, Ill.). We then added serially diluted mouse serum specimens to the wells and incubated them at 37°C for 90 minutes. After washing the wells with 1× PBST to discard uncaptured antibodies, we added negative COS-1 cell antigens, GI WT VLPs, GI KD VLPs, or GI GKD VLPs to the wells, which interacted with the captured IgG antibodies at 4°C overnight. On the following day, VLPs were recognized by rabbit anti-JEV polyclonal antibodies at 37°C for one hour, and the VLP-captured antibodies reacted with peroxidase-conjugated goat anti-rabbit IgG (H + L) (Jackson ImmunoResearch, West Grove, PA) at 37°C for another 1 hour. We described the steps of coloring and OD detection in Ag-ELISA. We calculated the ELISA endpoint titers of mice sera as the reciprocal dilution reaching a P/N ratio equal to three in sigmoidal dose-response regression of GraphPad Prism version 5.01. We calculated the total IgG titer by the endpoint titer of sera against WT, KD, and GKD VLPs for GI WT VLP-, GI KD VLP-, or GKD VLP-immunized mice. The DII_FL_ 106/107 IgG titer was calculated by subtracting the endpoint titer of sera against KD VLPs or WT VLPs from the total IgG titer for GI WT VLP- or KD VLP-immunized mice, respectively. The DII_FL_ 101/106/107 IgG titer was calculated by subtracting the endpoint titer of sera against GKD VLPs or WT VLPs from the total IgG titer for GI WT VLP- or GKD VLP-immunized mice, respectively. We gave the endpoint titer below the cut-off dilution of 400 an arbitrary value of 200.

### Focus-reduction micro-neutralization titer assay

We used the focus-reduction micro-neutralization titer (FRμNT) assay to measure the neutralizing activity of mice serum samples as described previously (91). Briefly, we seeded 2.25 × 10^4^ Vero cells on 96-well plates and incubated them for 18– 20 hours in a 37℃ incubator with 5% CO_2_. The mice sera were inactivated at 56℃ for 30 minutes and serially diluted. The 100 focus-forming units of the virus were incubated with the serial dilutions of mice sera at 37℃ for 1 hour. The serum-virus mixture infected the cells at 37℃ for another hour. The infected cells were then overlaid with 1% methylcellulose in DMEM with 2% FBS and incubated in a 37℃ incubator with 5% CO_2_ for 30 hours (JEV and WNV) or 72 hours (DENV2). The wells were washed with 1× PBS to remove the overlaid reagents and then fixed with 75% acetone at room temperature for 20 minutes. Mouse anti-JEV, WNV, or DENV2 HIAF bound to the virus antigens in the fixed and dried cells at 37℃ for 40 minutes, and peroxidase-conjugated goat anti-mouse IgG (Jackson ImmunoResearch, West Grove, PA) captured the bound antibodies at 37℃ for another 40 minutes. The foci were observed after adding the Vector-VIP peroxidase substrate kit SK-4600 (Vector Laboratories, Burlingame, CA). The images of foci were acquired by IMMUNOSPOT S6 Universal Analyzer (CELLULAR TECHNOLOGY). We calculated the FRμNT_50_ titer as the reciprocal dilution reaching a 50% reduction in fuci number compared to the virus control in sigmoidal dose-response regression of GraphPad Prism version 8. We assigned an arbitrary value of 5 to the FRμNT_50_ titer below the cut-off value of 10 for calculating the geometric mean titer (GMT).

### Depletion of VLP-reactive antibodies from the mouse serum specimen

Individual mouse serum used for the depletion study was calculated and diluted to have an eightfold higher end-point FRμNT_50_ serum titer. The diluted mouse serum was incubated with 5 × 10^6^ COS-1 cells transfected with VLP-expressing plasmids for an hour at 37℃. After incubation, the VLP-reactive antibodies were captured by the transfected cells, and the remaining antibodies, namely the VLP-reactive depleted serum specimen, were collected in the supernatant. We subsequently measured the neutralizing activity of the VLP-depleted sera in the FRμNT assay. The percentage of focus reduction was calculated for COS-1 cell control-depleted, WT VLP-depleted, KD VLP-depleted, and GKD VLP-depleted sera compared to 100% of focus reduction for the serum sample without depletion.

### The construction and generation of recombinant and mutant GI JEVs

An infectious clone encoding the full genome of the GI YL2009-4 virus (pCMV GI WT) was previously established (92). The introduction of DII_FL_ mutation(s) into pCMV GI WT was performed through PCRs (using KOD Hot Start DNA Polymerase from Merck, United States) with the primers listed in Table 2. The PCR treated with DpnI enzyme was then transferred into competent cells. Mutated clones were extracted from the transformed competent cells using a Mini-prep kit (Qiagen) and sequenced to confirm the complete viral genome insert. Recombinant GI JEV was generated as previously described (92). Briefly, BHK-21 cells were transfected with a mixture of 1 µg of the infectious clone, Opti-MEM (Life Technologies), and Lipofectamine 2000 (Life Technologies). After transfection for 5 hours at 37℃, the cell supernatant was replaced with the culture medium. Following a 3- to 4-day incubation, recombinant viruses secreted from transfected cells were harvested and subsequently amplified twice in BHK-21 cells. The production of rJEVs was detected in transfected cells using an immunofluorescence assay (IFA) with mouse anti-JEV HIAF. Virus plaques were identified by micro-plaque assay. Viral RNA was extracted from the supernatant of virus-infected BHK-21 cells using the RNeasy mini kit (Qiagen) and transcribed into cDNA with the JEV 3′UTR primer 5′-AGATCCTGTGTTCTTCCTCA-3′ using the Superscript III transcription reaction (Thermo Fisher Scientific). The full length of the JEV E gene was amplified by PCR with a pair of GI JEV E primers (5′-CGTGTGGTaTTCACTATTCTC-3′ and 5′-CATTCAGTTCGTCCCGCACA-3′).

### Statistical analysis

We compared the antibody responses between GI WT VLP-, KD VLP-, and GKD VLP-immunized mice by one-way ANOVA with Tukey’s multiple comparisons test. The comparison between the two groups was analyzed by a two-tailed unpaired *t*-test. We analyzed the survival curve of PBS, GI WT VLP-, and GI GKD VLP-immunized mice using the Log-rank test. Statistical significance was recognized as *P* ˂0.05. All these statistical analyses were conducted in GraphPad Prism version 8.

## Data Availability

The PDB templates of 3P54 and 5N0A were used to model the E dimers of the JEV GI YL2009-4 virus (accession number: JF499808.1) (83).
